# Beneficial impact of indocyanine green fluorescence imaging on lymphadenectomy in laparoscopic total gastrectomy for advanced upper gastric cancer

**DOI:** 10.3389/fonc.2025.1588048

**Published:** 2025-11-27

**Authors:** Chen-Bin Lv, Lin-Yan Tong, Yu-Qin Sun, Rong-Jie Huang, Huang-Huang Han, Qiu-Xian Chen, Li-sheng Cai

**Affiliations:** 1 Department of Gastric Surgery, Zhangzhou Affiliated Hospital of Fujian Medical University, Zhangzhou, Fujian, China; 2 Department of Ultrasound, Zhangzhou Affiliated Hospital of Fujian Medical University, Zhangzhou, Fujian, China; 3 Department of Statistics, Zhangzhou Affiliated Hospital of Fujian Medical University, Zhangzhou, Fujian, China

**Keywords:** indocyanine green, gastric cancer, fluorescence laparoscopy, lymph node dissection, prognosis

## Abstract

**Objective:**

This study aims to analyze the benefits of indocyanine green (ICG) fluorescence imaging on the efficacy of lymph node dissection (LND) during laparoscopic total gastrectomy (LTG) for advanced upper gastric cancer.

**Materials and methods:**

We retrospectively analyzed the clinicopathological data of 98 patients with advanced upper gastric cancer undergoing LTG, including 29 patients in the ICG-guided group and 69 in the conventional LTG (non-ICG) group. The perioperative outcomes, efficiency of LND, and survival outcomes were compared between the two groups.

**Results:**

The mean number of lymph nodes (LNs) dissected was greater in the ICG group than the non-ICG group (52.34 vs. 37.38; P < 0.001). Additionally, the ICG group had more patients with > 30 dissected LNs (96.55% vs. 76.81%; P = 0.018). Notably, the ICG group exhibited a higher number of LNs dissected at stations 7, 8, 9, and 11 than the non-ICG group (P < 0.05). Metastatic LNs were more frequently identified among fluorescence-positive LNs (P = 0.002). ICG fluorescence imaging demonstrated excellent diagnostic performance for metastatic LNs with a sensitivity of 85.9% and a negative predictive value of 96%. The ICG and non-ICG groups showed comparable 2-year overall survival (86.2% vs 82.6%, p=0.737) and disease-free survival (82.8% vs 72.5%, p=0.203) rates.

**Conclusions:**

ICG fluorescence imaging significantly improved lymphadenectomy precision during LTG for advanced upper gastric cancer, particularly in suprapancreatic nodal stations, and enhanced detection of metastatic LNs. However, no obvious survival benefit was observed within the limited follow-up period. Future prospective, multicenter studies are warranted to validate these results.

## 1 Introduction

Gastric cancer is a significant health burden in China, with a high incidence rate and poor prognosis, as the majority of patients are diagnosed at advanced stages (1). Surgical intervention remains a cornerstone of the management of advanced gastric cancer. In particular, the use of laparoscopic radical total gastrectomy (LTG) has increased owing to its minimal invasiveness, enabling rapid recovery (2, 3). However, advanced gastric cancer in the upper stomach requiring LTG poses a distinct surgical challenge. This procedure involves not only standard suprapancreatic lymph node dissection but also necessitates clearance of stations specific to the gastric fundus and proximal anatomy. These steps are technically demanding and carry increased risks of complications such as pancreatic fistula or splenic vessel injury. Consequently, a thorough lymphadenectomy in LTG carries a high risk of incomplete nodal retrieval, especially in these critical zones. This incompletion may, in turn, compromise oncologic outcomes.

With the evolution of precision medicine and surgical advancements, indocyanine green (ICG) near-infrared imaging has emerged as a valuable tool for laparoscopic gastric cancer surgery. This technology enables real-time visualization of lymphatic drainage and lymph node (LN) locations, providing surgeons with intuitive navigational information that may lead to more thorough lymph node dissection (LND) and improved surgical efficacy (4, 5). However, existing literature on ICG navigation in gastric cancer often amalgamates results from diverse tumor locations and surgical procedures. This heterogeneity may obscure its precise value in specific, high-risk scenarios. Despite the promising results, there is an ongoing debate regarding which lymph node regions or patient populations benefit the most from ICG-guided dissection, alongside unresolved debates regarding its prognostic implications (6).

In light of these considerations, the present study aimed to provide a comprehensive analysis of the efficacy of ICG in lymphadenectomy and its application to the diagnosis of metastatic lymph nodes during LTG for advanced gastric cancer. Furthermore, this study delved into the prognostic implications of ICG-guided lymphadenectomy, ultimately offering granular clinical evidence to optimize precision oncology strategies in gastric cancer management.

## 2 Materials and methods

### 2.1 Patients

A total of 179 patients with advanced upper gastric cancer who underwent LTG at the Department of Gastric Surgery, Affiliated Zhangzhou Hospital of Fujian Medical University from June 2021 to December 2022 were enrolled in this study. All included cases were operated by the same surgical team, with procedures performed by a senior surgeon (>300 laparoscopic gastrectomies) proficient in ICG near-infrared fluorescence imaging.

The inclusion criteria were as follows: age between 16 and 80 years; D2 lymph node dissection; postoperative pathology-confirmed gastric adenocarcinoma; and pathological staging of pT2-4a, N0-3, or M0. The exclusion criteria included the presence of distant metastasis, a history of gastric surgery (including endoscopic treatment for gastric cancer), prior neoadjuvant chemotherapy or radiotherapy, combined organ resection or thoracic surgery, linitis plastica, or missing clinical or pathological data. All enrolled patients received 4–6 cycles of standardized adjuvant chemotherapy (SOX or S1 regimen).

Patient assignment to the ICG or non-ICG group was determined clinically. ICG fluorescence navigation was routinely recommended for all eligible patients. Its final application depended on informed patient/family consent, the availability of the fluorescence imaging system, and intraoperative findings. Ultimately, 98 patients were included in the study and divided into the following two groups based on the surgical approach: ICG fluorescence imaging group (29 cases) and non-ICG fluorescence imaging group (69 cases). This study was approved by the Medical Ethics Committee of the Affiliated Zhangzhou Hospital, Fujian Medical University(Approval No. 2024LWB425). Informed consent was obtained from all subjects and/or their legal guardian(s).

### 2.2 Surgical procedures

The ICG injection protocol was standardized and identical for all patients in the ICG group. ICG was used as a tracer agent intraoperatively, following the recommended serosal injection method and pattern (7). ICG (25 mg/vial, diluted to 0.5 mg/mL with sterile water for injection) was injected at three points on the lesser and greater curvatures of the stomach, with 1.5 mL administered subserosally at each point, 20 min before LND. On the lesser curvature, injections were performed at the first branch of the right gastric artery, the angular incisure, and the midpoint between the first and second gastric wall branches of the left gastric artery. On the greater curvature, injections were performed at the first gastric branch of the right gastroepiploic artery, the first gastric branch of the left gastroepiploic artery, and the junction of the gastric fundus and body. The recommended injection points are depicted in **supplementary Figure 1**. In case the injection point is invaded by the tumor, the tumor margin is suggested as the injection point. The fluorescence mode was switched for navigation during LND (**supplementary Figure 2**). Dissection of the splenic hilar lymph nodes was reserved for tumors involving the upper gastric greater curvature or cases with suspected nodal metastasis on preoperative computed tomography imaging/intraoperative findings.

ICG was not used in the non-ICG group, and conventional laparoscopic gastric cancer radical surgery was performed according to the Japanese Gastric Cancer Treatment Guidelines (8). To achieve accurate tumor N staging, lymph nodes were sorted by the operating surgeon based on their location before pathological examination. In the ICG group, specimens were examined based on the ICG system product fluorescence after removal. Lymph nodes displaying fluorescence and those not displaying fluorescence were separately packaged, fixed with 10% formalin, and sent for pathological examination.

### 2.3 Observation items and definitions

Intraoperative blood loss was estimated using the suction and gauze weight method. The time to first ambulation was recorded when patients stood and walked at least 5 m. The time to first flatus was confirmed based on patient reports and auscultation by medical staff. The time to first oral intake was noted when patients consumed food orally for the first time. Postoperative hospital stay was measured from the end of surgery to the time of discharge.

The diagnostic performance of ICG fluorescence for metastatic lymph nodes (LNs) was evaluated per node. Each harvested LN in the ICG group was intraoperatively classified as fluorescent or non-fluorescent. Postoperative histopathology served as the gold standard, defining the following categories: True Positive (TP: fluorescent & metastatic), False Positive (FP: fluorescent and benign), True Negative (TN: non-fluorescent and benign), and False Negative (FN: non-fluorescent and metastatic). Sensitivity was calculated as TP/(TP+FN). Specificity was the proportion of non-metastatic LNs correctly identified as non-fluorescent (TN/[TN+FP]). The positive predictive value (PPV) was the proportion of fluorescent LNs that were metastatic (TP/[TP+FP]), and the negative predictive value (NPV) was the proportion of non-fluorescent LNs that were non-metastatic (TN/[TN+FN]). The Youden index was calculated as (Sensitivity + Specificity - 1).

Postoperative surgical complications, mainly related to the surgical system, included incisional infections, gastrointestinal leaks, postoperative bleeding, abdominal infections, and gastrointestinal obstructions (9), recorded within 1 month and graded according to the Clavien–Dindo classification system were included in analyses (10).

Overall Survival (OS) was defined as the time from surgery to death from any cause. Disease-Free Survival (DFS) was defined as the time from surgery to tumor recurrence or death from any cause, whichever came first.

### 2.4 Statistical analysis

Data were analyzed using SPSS 27.0 software (IBM, Armonk, NY, USA). Normally distributed continuous data are expressed as the mean ± standard deviation, and intergroup comparisons were performed using independent-samples Student’s t-tests. Data that were not normally distributed were analyzed using non-parametric tests. Categorical data are expressed as numbers and percentages, and intergroup comparisons were performed using the chi-squared test or Fisher’s exact probability test. Survival rates were analyzed using the Kaplan-Meier method to construct survival curves, followed by log-rank testing for between-group comparisons of time-to-event data. Statistical significance was set at P < 0.05.

## 3 Results

### 3.1 Patient characteristics

As shown in **Table 1**, there were no differences in age, sex, body mass index (BMI), tumor size, degree of differentiation, neural invasion, vascular invasion, pT stage, pN stage, or pTNM stage between the ICG and non-ICG groups (all P > 0.05).

**Table 1:** Characteristics of patients in the ICG and non-ICG groups.

Variables, n (%)	Total (n=98)	Non-ICG (n=69)	ICG (n =29)	P value
Sex				0.919
Female	23 (23.47)	16 (23.19)	7 (24.14)	
Male	75 (76.53)	53 (76.81)	22 (75.86)	
Age, yrs				0.536
<65	62 (63.27)	45 (65.22)	17 (58.62)	
≥65	36 (36.73)	24 (34.78)	12 (41.38)	
BMI, kg/m2				0.821
<25	84 (85.71)	60 (86.96)	24 (82.76)	
≥25	14 (14.29)	9 (13.04)	5 (17.24)	
Tumor size, mm				0.470
<50	32 (32.65)	21 (30.43)	11 (37.93)	
≥50	66 (67.35)	48 (69.57)	18 (62.07)	
Tumor grade				0.339
Differentiated	41 (41.84)	31 (44.93)	10 (34.48)	
Undifferentiated	57 (58.16)	38 (55.07)	19 (65.52)	
Lymphovascular Invasion				0.513
No	26 (26.53)	17 (24.64)	9 (31.03)	
Yes	72 (73.47)	52 (75.36)	20 (68.97)	
Neural Invasion				0.478
No	12 (12.24)	10 (14.49)	2 (6.90)	
Yes	86 (87.76)	59 (85.51)	27 (93.10)	
pT stage				0.488
T2	17 (17.35)	14 (20.29)	3 (10.34)	
T3	29 (29.59)	20 (28.99)	9 (31.03)	
T4	52 (53.06)	35 (50.72)	17 (58.62)	
pN stage				0.668
N0	31 (31.63)	22 (31.88)	9 (31.03)	
N1	22 (22.45)	16 (23.19)	6 (20.69)	
N2	22 (22.45)	17 (24.64)	5 (17.24)	
N3	23 (23.47)	14 (20.29)	9 (31.03)	
pTNM stage				0.577
I	12 (12.24)	10 (14.49)	2 (6.90)	
II	29 (29.59)	20 (28.99)	9 (31.03)	
III	57 (58.16)	39 (56.52)	18 (62.07)	

ICG, Indocyanine green; BMI, body mass index.

### 3.2 Perioperative outcomes

The ICG and non-ICG groups showed no differences in intraoperative blood loss, time to first ambulation, time to first flatus, time to first oral intake, or postoperative hospital stay (all P > 0.05). The overall postoperative surgical complication rate was 19.64%, with estimates of 21.74% in the non-ICG group and 17.24% in the ICG group (P = 0.614). The majority of complications were grade II according to the Clavien-Dindo classification system, with postoperative infections and bleeding being the most common types. The incidence and severity of complications were comparable between the two groups (P > 0.05; **Table 2**).

**Table 2:** Perioperative outcome for ICG and non-ICG groups.

Variables, mean ± SD/n (%)	Total (n=98)	Non-ICG (n=69)	ICG (n =29)	P value
Operative time (min)	227.94 ± 33.84	224.14 ± 35.25	236.97 ± 28.81	0.087
Blood loss (ml)	65.84 ± 44.05	70.27 ± 48.67	61.76 ± 39.38	0.343
Time to ambulation (days)	1.50 ± 0.60	1.53 ± 0.67	1.49 ± 0.57	0.676
Time to first flatus (days)	2.80 ± 0.90	2.92 ± 0.98	2.74 ± 0.86	0.239
Time to first liquid intake (days)	4.69 ± 1.74	4.59 ± 0.91	4.74 ± 2.00	0.694
Time to first semifluid intake (days)	6.37 ± 1.20	6.44 ± 1.38	6.20 ± 0.57	0.100
Postoperative hospital stay days	9.15 ± 3.98	9.32 ± 4.13	8.76 ± 3.64	0.528
Postoperative surgical complications	21 (19.64)	15 (21.74)	5 (17.24)	0.614
Wound infection	3 (3.06)	2 (2.90)	1 (3.45)	0.885
Lymphatic leakage	2 (2.04)	2 (2.90)	0 (0.00)	0.354
Anastomotic leakage	3 (3.06)	2 (2.90)	1 (3.45)	0.885
Anastomotic bleeding	2 (2.04)	1 (1.45)	1 (3.45)	0.523
Ileus	3 (3.06)	2 (2.90)	0 (0.00)	0.354
Abdominal bleeding	3 (3.06)	2 (2.90)	1 (3.45)	0.885
Duodenal stump fistula	1 (1.02)	1 (1.45)	0 (0.00)	0.515
Abdominal infection	4 (4.08)	3 (4.35)	1 (3.45)	0.837
Clavien-dindo classification				
I	2 (2.04)	1 (1.45)	1 (3.45)	0.523
II	11 (11.22)	8 (11.59)	3 (10.34)	0.858
IIIa	4 (4.08)	3 (4.35)	1 (3.45)	0.837
IIIb	2 (2.04)	2 (2.90)	0 (0.00)	0.354
IV	1 (1.02)	1 (1.45)	0 (0.00)	0.515
V	0 (0.00)	0 (0.00)	0 (0.00)	-

ICG, Indocyanine green.

### 3.3 Comparison of LND between the two groups

As shown in **Table 3**, the mean total number of LNs dissected was higher in the ICG group than in the non-ICG group (52.34 vs. 37.38; P < 0.001). The proportion of patients with > 30 dissected LNs was also higher in the ICG group (96.55% vs. 76.81%, P = 0.018). Additionally, greater numbers of LNs were retrieved from both the perigastric (33.41 vs. 26.65; P = 0.009) and extraperigastric (19.10 vs. 10.72; P < 0.001) regions in the ICG group than in the non-ICG group. Stratified analyses confirmed that the mean total number of nodes dissected remained higher in the ICG group than in the non-ICG group across various subgroups, including sex, BMI, tumor size, tumor grade, and pT, pN, and pTNM stages.

**Table 3:** Number of LNs dissected in ICG and non-ICG groups.

Variables, mean ± SD/n (%)	Total (n=98)	Non-ICG (n=69)	ICG (n=29)	P value
Total number of LNs dissected	41.81 ± 12.37	37.38 ± 9.54	52.34 ± 12.09	<.001
Number of LNs dissected ≥30	83 (84.69)	53 (76.81)	28 (96.55)	0.018
Perigastric LNs	28.65 ± 10.24	26.65 ± 8.74	33.41 ± 12.01	0.009
extraperigastric LNs	13.20 ± 7.18	10.72 ± 5.57	19.10 ± 7.22	<.001
Total number of LNM	4.63 ± 6.61	3.99 ± 5.24	6.17 ± 9.00	0.135
Sex				
female	44.57 ± 15.05	37.38 ± 10.13	61.00 ± 11.02	<.001
male	40.96 ± 11.41	37.38 ± 9.45	49.59 ± 11.28	<.001
BMI, kg/m2				
<25	41.71 ± 13.16	36.56 ± 7.54	51.00 ± 16.78	0.043
≥25	41.82 ± 12.32	37.50 ± 9.85	52.62 ± 11.34	<.001
Tumor size, mm				
<50	39.06 ± 10.55	35.38 ± 8.55	46.09 ± 10.78	0.004
≥50	43.14 ± 13.03	38.25 ± 9.90	56.17 ± 11.47	<.001
Tumor grade				
Differentiated	42.29 ± 11.06	38.74 ± 8.85	53.30 ± 10.21	<.001
Undifferentiated	41.46 ± 13.33	36.26 ± 10.04	51.84 ± 13.21	<.001
pT stage				
T2-3	41.26 ± 11.72	37.24 ± 8.68	52.67 ± 11.99	<.001
T4	42.29 ± 13.02	37.51 ± 10.43	52.12 ± 12.52	<.001
pN stage				
N0	40.65 ± 12.75	35.55 ± 7.69	53.11 ± 14.43	<.001
N+	42.34 ± 12.26	38.23 ± 10.25	52.00 ± 11.28	<.001
pTNM stage				
I	37.33 ± 8.32	35.10 ± 6.47	48.50 ± 9.19	0.029
II	42.52 ± 13.74	36.40 ± 9.29	56.11 ± 12.42	<.001
III	42.39 ± 12.34	38.46 ± 10.33	50.89 ± 12.31	<.001

ICG, Indocyanine green; LNs, lymph nodes; LNM, lymph node metastasis; BMI, body mass index.

**Figure 1:**
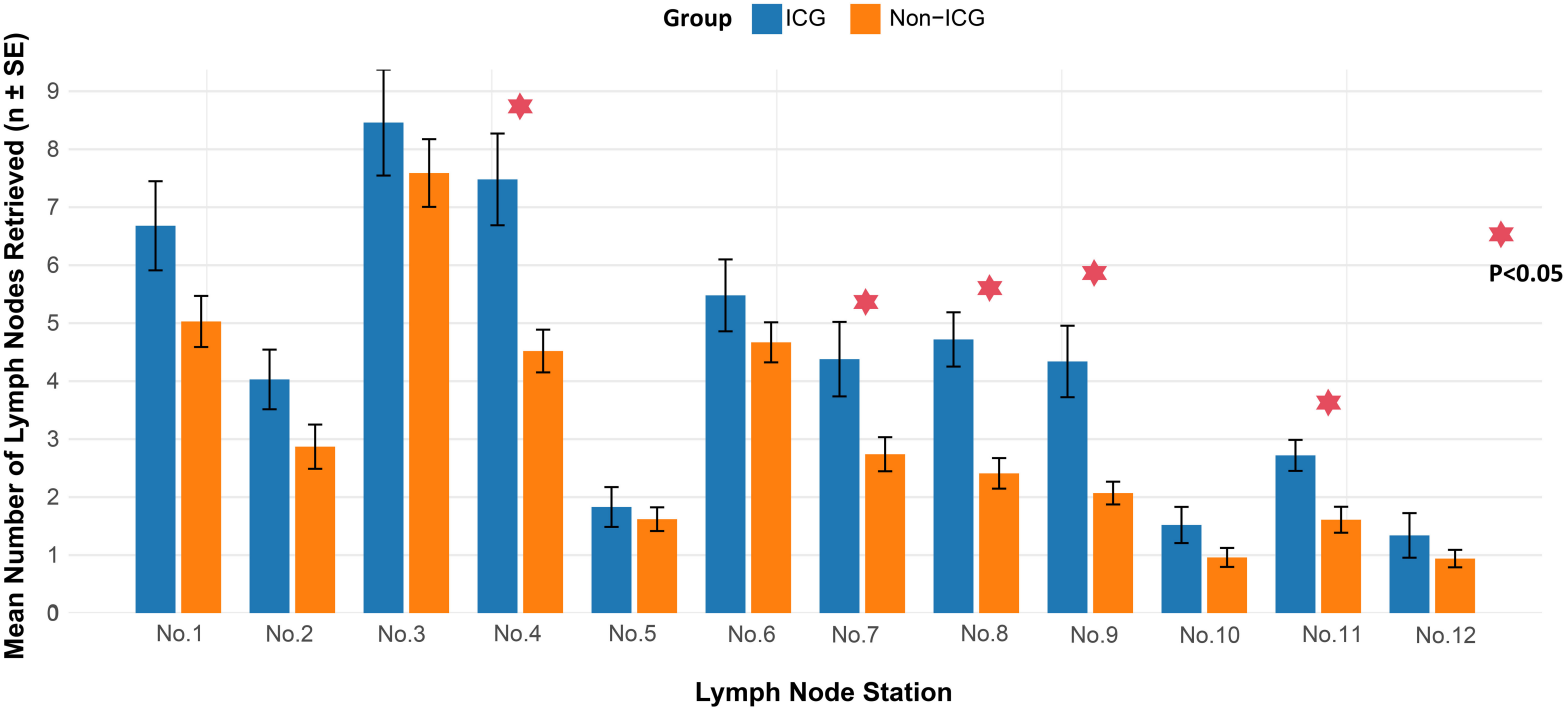
Comparison of mean lymph node dissection count in the ICG and non-ICG groups. SE, Standard Error. The asterisk (*) denotes a significant difference (p < 0.05).

Notably, at station 4 and the superior margin area of the pancreas (stations 7, 8, 9, and 11), the ICG group exhibited a greater number of LNs dissected than that in the non-ICG group (P < 0.05). (**Figure 1**).

### 3.4 Relationship between fluorescence and lymph node metastasis

**Figure 2:**
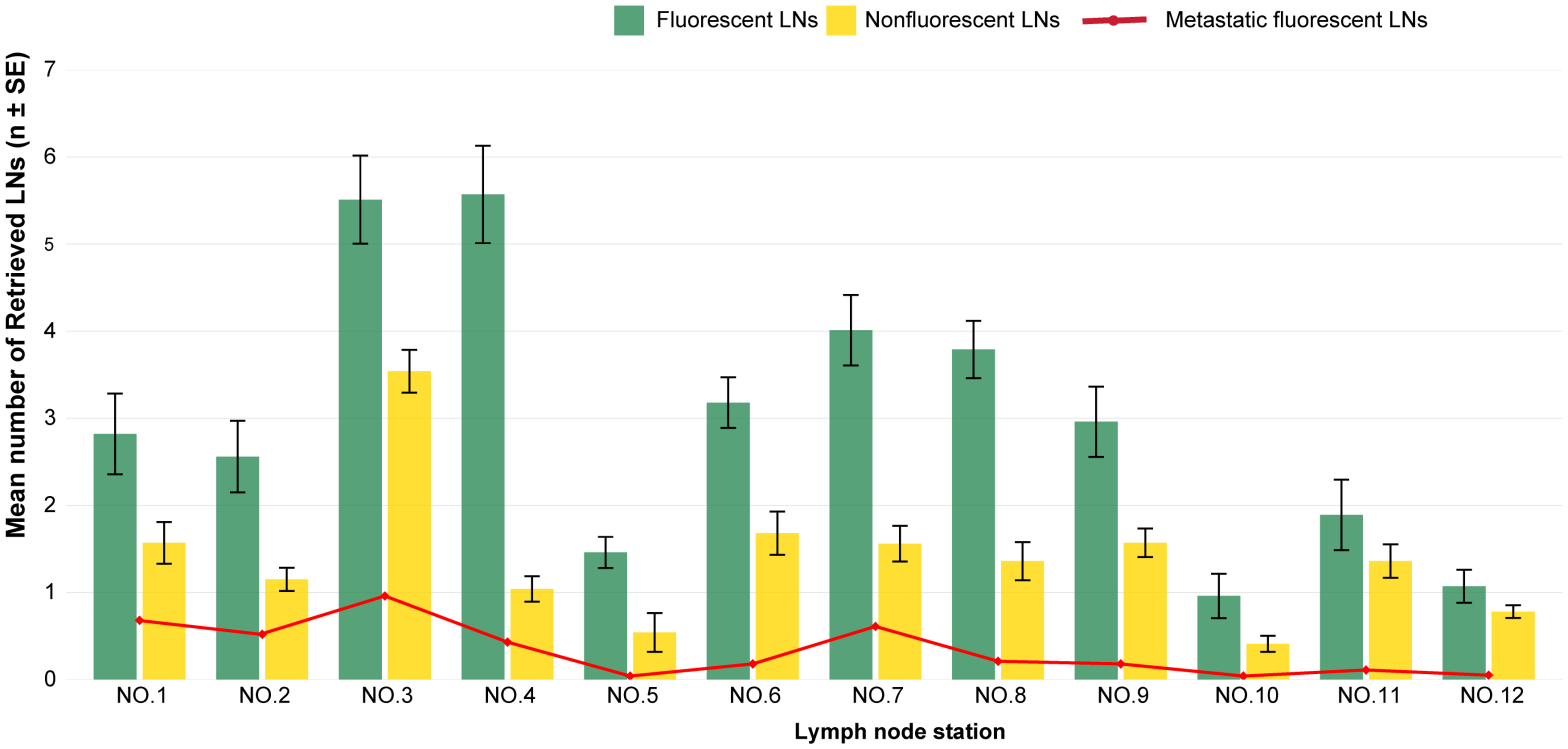
Comparison of mean fluorescent and non-fluorescent lymph node retrieval and number of metastatic fluorescents in ICG Group. SE, Standard Error.

Within the ICG group, the number of fluorescent LNs was greater than the number of non-fluorescent LNs (35.78 vs. 16.56; P < 0.001), and metastatic LNs were more frequently identified among fluorescent LNs (4.01 vs. 0.67; P = 0.002). In the stratified analysis according to LN station, the number of fluorescent LNs consistently exceeded the number of non-fluorescent LNs at each station, and metastatic fluorescent LNs were more frequently identified at stations 1, 2, 3 and 7. (**Figure 2**). ICG fluorescence imaging demonstrated excellent diagnostic performance for metastatic LNs, with a sensitivity of 85.9% and negative predictive value of 96% (**Supplementary Table 1**).

### 3.5 Comparative analysis of postoperative survival outcomes

**Figure 3:**
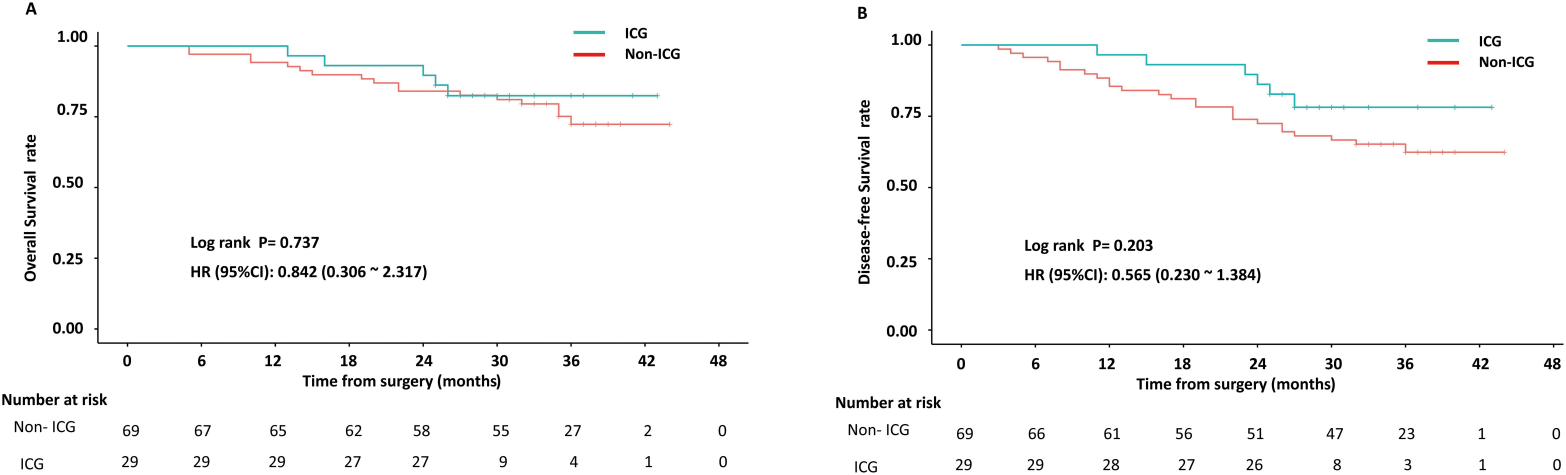
Comparison of survival outcomes in the ICG and non-ICG groups: **(A)** Overall survival; **(B)** Disease-free survival.

With the final follow-up date extending through February 28, 2025, the median follow-up duration for the entire cohort was 34 months (range: 26–44 months). Comparative analysis revealed comparable 2-year survival outcomes between groups: the ICG group demonstrated 86.2% OS and 82.8% DFS rates, while the non-ICG group achieved 82.6% OS and 72.5% DFS rates. These intergroup differences did not reach statistical significance (OS: p=0.737; DFS: p=0.203), as detailed in **Figure 3**.

## 4 Discussion

This study was designed to test the hypothesis that ICG fluorescence imaging provides significant value in ensuring a complete lymphadenectomy during LTG for advanced upper gastric cancer, a procedure characterized by unique technical challenges. Consistent with our focus on the technical difficulties of LTG, the data revealed that the benefit of ICG was not uniform but was particularly evident in key anatomical regions (Stations #7, #8, #9, and #11).

ICG is well-recognized for its safety profile, characterized by minimal toxicity and a low risk of allergic reactions. Most studies confirm that ICG navigation is both safe and feasible, demonstrating comparable short-term outcomes to conventional laparoscopic surgery without increasing postoperative complications (11, 12). In our study, the operative time, intraoperative blood loss, postoperative recovery, and postoperative surgical complication rates did not differ between the ICG and non-ICG groups (P > 0.05). The number of LNs dissected was significantly higher in the ICG group than the non-ICG group, while the mean number of LN metastases did not differ between the two groups. In a meta-analysis of 13 studies showed that, without increasing the operation time, intraoperative blood loss, or postoperative complications, more LNs were dissected in the ICG group than in the non-ICG group (P < 0.001) (13). Similarly, in the FUGES-012 prospective study, more LNs were dissected in the ICG group than in the non-ICG group (50.5 vs. 42.0, P < 0.01), with comparable postoperative recovery and complication rates between the two groups (14). However, Lan et al. (15) found that the ICG group did not result in an increase in the total number of nodes dissected, and this difference among studies may be related to differences in ICG injection methods and concentrations.

Accurate N staging requires dissection of at least 16 LNs, with 30 or more LNs being optimal (8). In our study, all patients had more than 16 LNs dissected, and the proportion of patients with more than 30 dissected LNs was higher in the ICG group than the non-ICG group, indicating that ICG helps identify missed lymphatic tissue and increases the number of LNs dissected. Kwon et al. (16) found that the ICG resulted in a significantly higher number of nodes dissected at stations 2, 6, 7, 8, and 9. Lee et al. (17) reported that the ICG group had an advantage in LNs dissected at station 10. The divergent patterns of lymph node enhancement observed across studies may be attributed to several factors. A key methodological difference is the ICG injection technique; the endoscopic submucosal injection used in the cited studies (16, 17) targets a different lymphatic drainage initiation point compared to the subserosal approach employed in our work, which could lead to variations in the primary lymphatic basins highlighted. Furthermore, differences in patient populations and proficiency in interpreting ICG fluorescence likely also contribute to the observed variances. Our study found that more LNs were retrieved in the ICG group, particularly in the upper pancreatic region (stations 7, 8, 9, 11). This is likely attributable to the area’s complex anatomy, which features abundant lymphatic and vascular adipose tissue with ill-defined planes to the pancreas. In such cases, ICG imaging can effectively guide and improve the dissection precision. Additionally, a stratified analysis confirmed that the mean total number of nodes dissected remained higher in the ICG group than in the non-ICG group across various subgroups (e.g., sex, BMI, tumor size, tumor grade, and pT, pN, and pTNM stage), consistent with previous results (14, 18). Furthermore, we found that the number of fluorescent LNs was higher than the number of non-fluorescent LNs, and the fluorescent LNs had a higher mean number of metastatic LNs than did the non-fluorescent LNs. The diagnostic sensitivity of ICG imaging for metastatic LNs was 85.9%, which is consistent with the reported range of 52.6–95.3% (19, 20). This indicates that fluorescent LNs receive lymphatic drainage from the tumor surroundings, but may not necessarily have cancer metastasis. The negative predictive value of nonfluorescent lymph nodes in our study was 96.0%; the false-negative rate was non-zero, consistent with that in previous studies (21, 22). This suggests that the lymphatic return pathways of some metastatic LNs were completely blocked by cancer cells, thereby preventing ICG backflow. However, the notably low specificity (33.4%) and Youden Index (0.19) indicate a high false-positive rate, meaning that a positive fluorescence signal is not a reliable predictor of metastasis. This underscores that fluorescence can be triggered by factors other than cancer cells, such as inflammation or macrophage accumulation in benign nodes. Therefore, the principal clinical value of ICG lies in its role as an exceptional lymphatic mapping and retrieval tool to guide a thorough lymphadenectomy, rather than as an intraoperative diagnostic test for metastasis. Surgeons should utilize ICG to enhance lymph node harvest completeness but must continue to depend on definitive postoperative histopathology for nodal staging.

Emerging evidence suggests that more extensive lymphadenectomy may confer survival benefits in long-term follow-up, yet data regarding the prognostic impact of ICG-guided radical gastrectomy remain scarce. Huang et al. conducted a prospective clinical trial demonstrating that ICG-navigated laparoscopic gastrectomy significantly improved 3-year OS (86.0% vs 73.6%, p=0.015) and DFS (81.4% vs 68.2%, p=0.012) rates compared to conventional surgery (23). However, a Spanish propensity score-matched study revealed comparable 2-year OS and DFS rates between ICG and non-ICG groups (24). Intriguingly, our study similarly demonstrated no statistically significant differences in 2-year OS ((86.2% vs 82.6%, p=0.737) or DFS (82.8% vs 72.5%, p=0.203) between cohorts. Furthermore, by strictly enrolling only patients with upfront surgery who completed standardized adjuvant chemotherapy, we aimed to isolate the effect of ICG navigation on surgical quality and intermediate outcomes, reducing the confounding impact of heterogeneous systemic therapy. However, the interpretation of long-term survival trends is constrained by the progressive reduction in the number of patients at risk beyond 30 months (**Figure 3**), which limits the statistical reliability of the Kaplan-Meier estimates in the later follow-up period. Therefore, the long-term trajectories of the survival curves should be interpreted with caution. The divergent outcomes across studies can be attributed to multiple factors: inherent patient heterogeneity; variations in surgical protocols, especially in operator-dependent factors like surgical skill and ICG mastery; and methodological inconsistencies in follow-up and assessment. Notably, The current evidence may underestimate true clinical outcomes because the non-ICG group’s median lymph node retrieval exceeds 30—a benchmark for adequate dissection. This suggests that while ICG enhances dissection, the non-ICG group’s lymph node retrieval quality may already be sufficient. Thus, further improvements in lymphadenectomy are unlikely to affect survival outcomes.

Looking forward, the future of fluorescence-guided precision surgery in gastric cancer lies in overcoming the specificity limitations inherent to non-targeted agents like ICG. While ICG serves as an excellent lymphatic mapping tool, as demonstrated by its high sensitivity, its low specificity for metastasis underscores the need for more selective targeting strategies. The emerging field of tumor-specific molecular imaging aims to address this precise challenge. The development of fluorescent probes targeting tumor-specific biomarkers—such as carcinoembryonic antigen-related cell adhesion molecule 5 (CEACAM5), human epidermal growth factor receptor 2 (HER2), or other gastric cancer-associated antigens—holds the potential to truly revolutionize surgical oncology (25). Such targeted agents could enable the visual intraoperative discrimination of metastatic from non-metastatic lymph nodes. This would shift the paradigm from a thorough, anatomy-based lymphadenectomy guided by non-targeted mapping to a precision resection that specifically preserves non-metastatic nodes.

Our study has several important limitations that should be acknowledged. First, its retrospective design and conduct at a single institution carry an inherent risk of selection bias and may limit the generalizability of our findings to broader populations with different surgical protocols and patient demographics. Second, although based on existing literature, the ICG administration and imaging protocol lacked universal standardization, potentially introducing variability in fluorescence signals. Third, the modest sample size and significant group imbalance (ICG: 29 vs. Non-ICG: 69) are important limitations. This imbalance renders the study potentially underpowered, increasing the risk of Type II errors wherein clinically meaningful survival benefits might have been undetected. Finally, the relatively short follow-up period precludes definitive conclusions on long-term outcomes. Therefore, our findings should be considered exploratory and validated through prospective, multicenter, randomized controlled trials.

## 5 Conclusion

In conclusion, ICG fluorescence imaging demonstrated superior efficacy in optimizing lymph node dissection precision during LTG for advanced upper gastric cancer, particularly at anatomically challenging suprapancreatic nodal stations. Notably, its ability to intraoperatively pinpoint LNs at higher risk for metastasis serves as a valuable adjunct to conventional surgical protocols. Nevertheless, the technique did not demonstrate significant survival advantages within the current observation period. Further investigation with extended follow-up is warranted to determine whether these technical improvements may translate into measurable long-term oncological benefits.

## Data Availability

The original contributions presented in the study are included in the article/**Supplementary Material**. Further inquiries can be directed to the corresponding authors.

## Supplementary material

Supplementary Figure 1 The recommended injection points for subserosal injection of ICG.

Supplementary Figure 2 ICG fluorescence imaging sample pictures during lymph node dissection. **(A, B)**: Fluorescent lymph nodes; **(C)**: Fluorescent lymphatic vessels; **(D)**: Exposed right gastroepiploic vein after the lymphoid tissue was dissected.
