# Supplementary material for: Beneficial impact of indocyanine green fluorescence imaging on lymphadenectomy in laparoscopic total gastrectomy for advanced upper gastric cancer
Source: Front Oncol. 2025 Nov 27;15:1588048. doi: 10.3389/fonc.2025.1588048 (PMC12695527; doi:10.3389/fonc.2025.1588048)
Supplement: Supplementary file 1 [file Table1.docx]

**Supplementary Table 1.** The validity related to ICG that emitted fluorescence and lymph node metastasis

| **Variables** | Value |
| --- | --- |
| **Sensitivity** | 85.9%(116/135) |
| **False negative rate** | 14.1%(19/135) |
| **Specificity** | 33.4%(461/1382) |
| **False positive rate** | 66.6%(921/1382) |
| **Positive likelihood ratio** | 1.29 |
| **Negative likelihood ratio** | 0.42 |
| **Positive predictive value** | 11.9%(116/1037) |
| **Negative predictive value** | 96.0%(461/480) |
| **Youden index** | 0.19 |

**Abbreviations:** ICG, Indocyanine green
