# Supplementary figures and images for: Beneficial impact of indocyanine green fluorescence imaging on lymphadenectomy in laparoscopic total gastrectomy for advanced upper gastric cancer
Source: Front Oncol. 2025 Nov 27;15:1588048. doi: 10.3389/fonc.2025.1588048 (PMC12695527; doi:10.3389/fonc.2025.1588048)

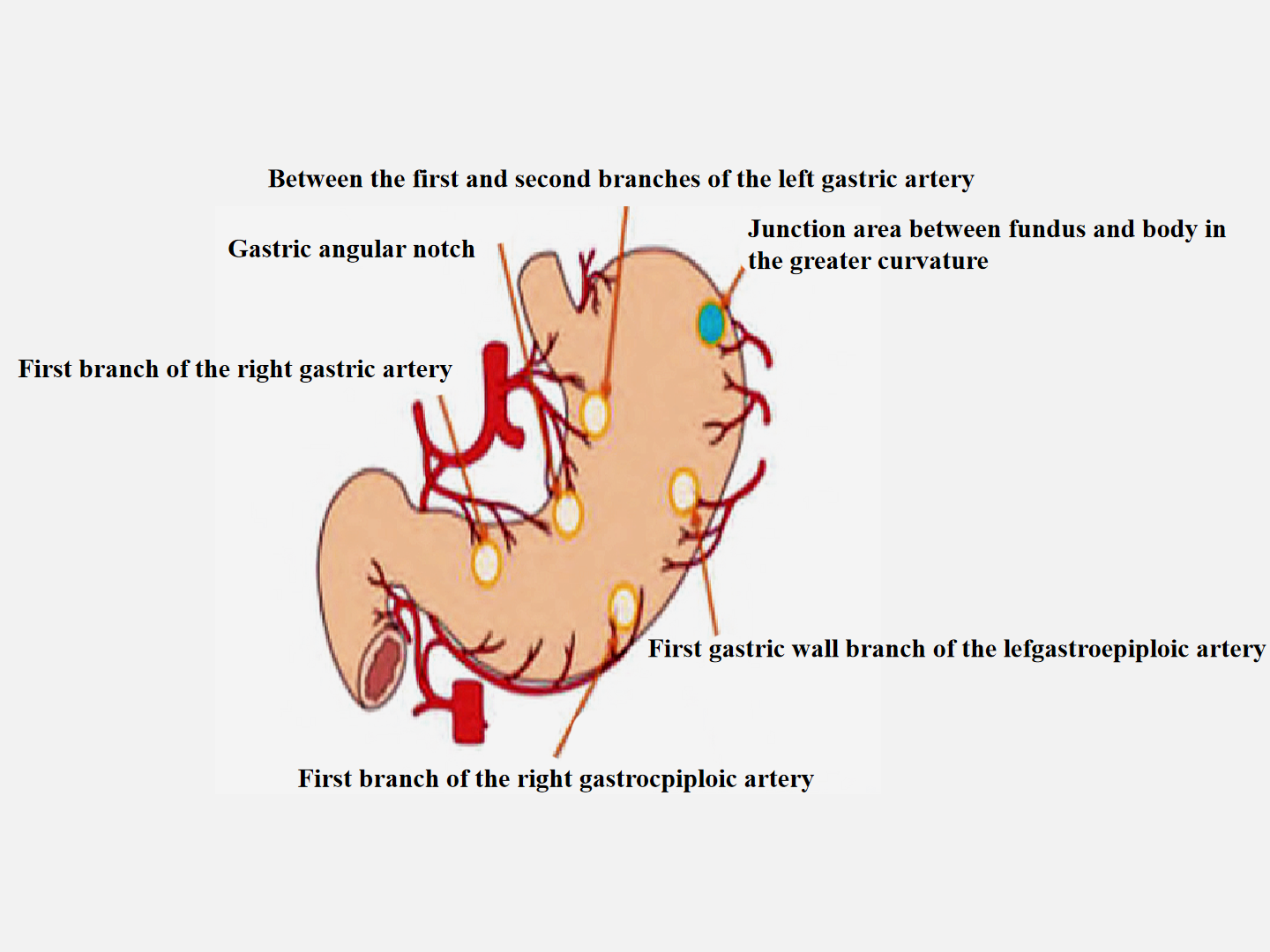

Supplement: Supplementary Figure 1 — The recommended injection points for subserosal injection of ICG. [file Image1.tif]

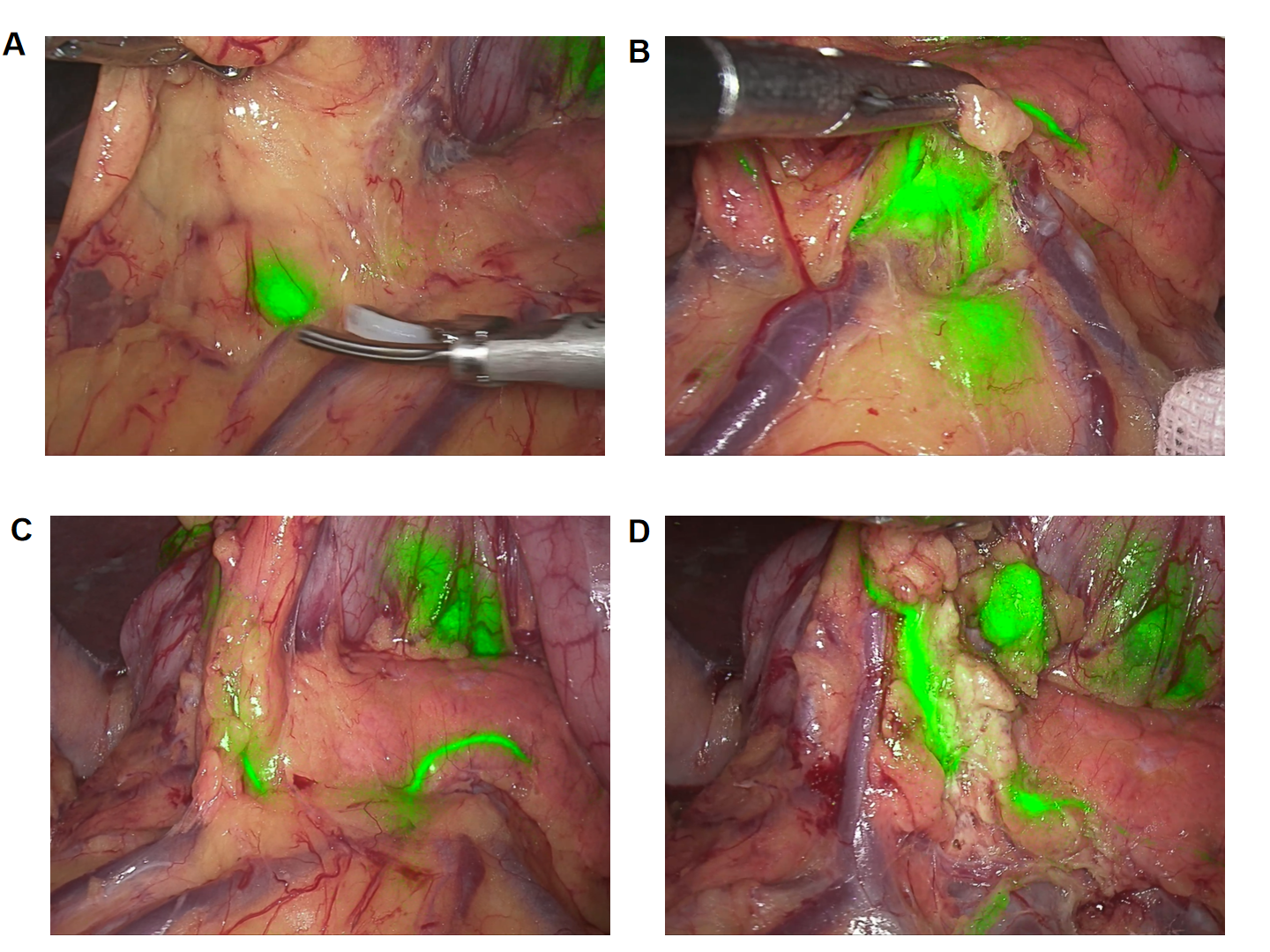

Supplement: Supplementary Figure 2 — ICG fluorescence imaging sample pictures during lymph node dissection. (A, B): Fluorescent lymph nodes; (C): Fluorescent lymphatic vessels; (D): Exposed right gastroepiploic vein after the lymphoid tissue was dissected. [file Image2.tif]
